# Neonatal point-of-care ultrasound—steps for program development

**DOI:** 10.1038/s41372-025-02359-1

**Published:** 2025-08-01

**Authors:** Shazia Bhombal, Yogen Singh, Stephanie Marshall, Shanmukha Mukthapuram, María V. Fraga

**Affiliations:** 1 Department of Pediatrics, Children’s Healthcare of Atlanta/Emory University, Atlanta, GA USA; 2 Department of Pediatrics, Division of Neonatology, University of California, UC Davis Children’s Hospital, Sacramento, CA USA; 3 Department of Pediatrics, Division of Neonatology, Ann & Robert H Lurie Children’s Hospital and Northwestern University Feinberg School of Medicine, Chicago, IL USA; 4 Department of Pediatrics, Nemours Children’s Health, Cleveland Clinic Tradition Hospital, Port St. Lucie, FL USA; 5 Department of Pediatrics, Children’s Hospital of Philadelphia and Perelman School of Medicine, University of Pennsylvania, Philadelphia, PA USA; 6 Children’s Healthcare of Atlanta/Emory University, Atlanta, GA USA; 7 Division of Neonatology, University of California, UC Davis Children’s Hospital, Sacramento, CA USA; 8 Division of Neonatology, Ann & Robert H Lurie Children’s Hospital and Northwestern University Feinberg School of Medicine, Chicago, IL USA; 9 Nemours Children’s Health, Cleveland Clinic Tradition Hospital, Port St. Lucie, FL USA; 10 Children’s Hospital of Philadelphia and Perelman School of Medicine, University of Pennsylvania, Philadelphia, PA USA; 11 Dell Medical School, University of Texas at Austin, Austin, TX USA; 12 University of Colorado School of Medicine, Denver, CO USA; 13 University of North Carolina, Chapel Hill, NC USA; 14 Baylor College of Medicine, Texas children’s Hospital, Houston, TX USA; 15 Children’s Hospital of Los Angeles, Keck School of Medicine, University of Southern Carolina, Los Angeles, CA USA; 16 Icahn School of Medicine at Mount Sinai, New York, NY USA; 17 University of California San Francisco, San Francisco, CA USA; 18 Orlando Health Hospital, Orlando, FL USA; 19 New York-Presbyterian Morgan Stanley Children’s Hospital, New York, NY USA; 20 University of Wisconsin School of Medicine and Public Health and UnityPoint Health Meriter, Madison, WI USA; 21 University of California Irvine School of Medicine, Irvine, CA USA

**Keywords:** Medical imaging, Paediatrics

## Abstract

Point-of-care ultrasound (POCUS) is increasingly being utilized in the neonatal field; however, there is a lack of standardized guidance on the development of POCUS programs in neonatology. While subspecialties with decades of POCUS experience have established pathways for program development and training guidelines, such frameworks are absent in neonatal and perinatal medicine. Recent national and international publications have highlighted the need for structured program development and have established recommendations regarding standards for POCUS use, including minimum number of scans, competency-based assessments, and a strong quality improvement (QI) framework. This publication aims to provide steps toward program development for neonatal intensive care units eager to establish POCUS to augment the care of their patients.

## Introduction

The recognition in critical care medicine that Point-of-care ultrasound (POCUS) enhances triage, improves diagnostic accuracy, and benefits patient care has resulted in the rapid adoption of bedside ultrasound by clinicians [1]. POCUS has been utilized for decades in adult subspecialties such as anesthesia and emergency medicine for diagnostic and procedural applications. In 2001, emergency medicine guidelines for the use of POCUS were established to standardize programmatic development and include POCUS training in emergency medicine curricula [2].

Although the field of pediatrics has been a late adopter of POCUS, literature outlining the benefits of ultrasound in neonates, including assessment of endotracheal tube and central line tip positioning, was published over a decade ago [3, 4]. Recently, the use of POCUS has surged in pediatric subspecialties, with numerous publications addressing its indications and limitations [5–8]. However, guidelines standardizing programmatic development and integration into training programs are not yet available.

In 2022, the American Academy of Pediatrics, Section of Neonatal and Perinatal Medicine, recognized the value of POCUS as an adjunct to clinical care and published a clinical and technical report [5]. While the authors outlined indications as well as steps for programmatic development, they recognized the novelty of use in the neonatal intensive care unit (NICU) and barriers including lack of training guidelines, access to equipment, and insufficient expert facilitators. The Neonatal National POCUS Collaborative (NNPC) was established in 2023 to coalesce efforts towards standardizing POCUS implementation in neonatology and aid in providing a roadmap for program development. The mission of the Collaborative is ‘…to provide structure, standardize training programs and programmatic platforms, foster interdisciplinary collaboration with pediatric cardiology and pediatric radiology to ensure safe implementation of these programs, and promote innovation in the use of POCUS in the Neonatal Intensive Care units to enhance clinical care for our fragile population and improve patient outcomes’ (https://www.neopocuscollab.com/). This publication outlines the steps necessary to establish a neonatal POCUS program.

## Programmatic needs

Successful development of a neonatal POCUS program involves the integration of 7 components: (1) defining  the scope of practice, (2) Identifying stakeholders, (3) formalizing the educational curriculum, (4) acquiring equipment, (5) outlining clinical workflow, (6) establishing documentation protocols, (7) implementing  a quality assurance and credentialing process. Each of these components may differ in development and maintenance based on institutional standards.

## 1) Scope of practice

POCUS is an adjunct to clinical care, providing time-sensitive information on a limited exam, refining differential diagnoses, and guiding clinical-decision making. Pediatric emergency medicine specialists have emphasized the role of POCUS as a valuable tool for rapid assessment, helping to streamline the triage process and accelerate patient management [9, 10].

Defining the scope of practice is a vital step in development of POCUS programs. Outlining basic and advanced POCUS applications in neonatology will provide a platform for further programmatic development including standardized training pathways. Neonatal POCUS scope of practice is dependent on local patient population, hospital structure, and clinical needs. This results in the identification of specific skills that will require structured training which will create a path to competence in practice.

## 2) Identification of stakeholders

POCUS program development requires the integration of key stakeholders to ensure successful implementation. Stakeholders include a NICU POCUS program director, providers from neonatology, cardiology, radiology, critical care and emergency medicine, divisional, and hospital leadership [5–11]. A need for a NICU program director was outlined in the AAP neonatal POCUS clinical report, who responsible for overseeing the development and maintenance of the program [5]. Ideally, the NICU program director should collaborate with the institutional credentialing body to incorporate POCUS into neonatal delineation of privileges, while also establishing a workflow to support billing, and the quality assurance (QA) process.

Non-NICU POCUS collaborators play a vital role in program development, as many pediatric specialties have already integrated POCUS into their standard of care. This is particularly evident in emergency medicine, where guidelines were published nearly a decade ago through collaborations with the AAP and emergency medicine societies [12]. Additionally, other POCUS-trained personnel within the hospital, such as anesthesia and pediatric intensive care specialists, can serve as valuable resources for educational support and contribute to shared infrastructure. Barriers identified in surveys for unsuccessful implementation of POCUS in the NICU included a perceived or actual lack of support from radiology and cardiology [13, 14]. However, cardiology’s recognition of the utility of POCUS has fostered greater opportunities for collaboration [6, 7]. Guidelines from the American Society of Echocardiography outline educational support for teaching cardiac POCUS [15]. This collaboration enhances educational support and quality improvement (QI) processes, with well-established metrics for assessment of image acquisition and interpretation [16].

## 3) Formalizing the educational curriculum

A key component in developing neonatal POCUS programs is structured training. While national guidelines for competency are unavailable, the literature supports a combination of didactic learning and hands-on experience for image acquisition and interpretation [17, 18]. A training pathway includes determining learning objectives, developing a curriculum, providing standardized scanning protocols, outlining expectations for image quality, interpretation, and competency standards. Basic and advanced applications of POCUS, as outlined by the NNPC in the accompanying publication, provide a framework for educational curricula. Integrating POCUS education within fellowship training and for faculty could be accomplished by ensuring participation in a 2-day POCUS “bootcamp”. This “bootcamp” encompasses didactic and hands-on sessions covering basic ultrasound principles and an overview of the scope of practice for bedside ultrasound [18–20].

Educational pathways include the development of competency specifications. Recognizing varied exposure to POCUS among learners, competency may be achieved at different rates. More fellows are exposed to ultrasound earlier in training, with medical schools and residency programs incorporating ultrasound education into their curricula [21, 22]. NICU POCUS training pathways may differ for a variety of learners, highlighting the  need to develop a pathway toward competency assessment [23]. However, there is still uncertainty and lack of knowledge about the best pathway to develop competency in neonatal POCUS.

## 4) Equipment acquisition

Each institution should have a dedicated, fit-for-purpose ultrasound machine to allow users to acquire and archive high-quality images. It is critically important that the ultrasound machine meets the institutional POCUS requirements and supports the imaging needs of neonates. Therefore, ultrasound machines should be equipped with a variety of probes including phased array and high-frequency linear probes.

When new equipment is introduced, an agreement should be sought at local level on:

a) *Maintenance of ultrasound machines and probes:* Institutional support is needed to ensure safety of ultrasound machine and integration with an image repository and documentation. Ultrasound equipment needs to be serviced regularly, according to the manufacturer’s recommendation.

b) *POCUS workflow:* Ultrasound machines should be able to allow smooth patient workflow, electronic documentation and the ability to archive images. POCUS study findings should be documented in patient’s charts and all images should be archived for appropriate reference and QA purposes.

c) *Local protocols:* Each unit should develop standard protocols for POCUS image acquisition based on national and international recommendations, available literature and use appropriate strategies to incorporate POCUS workflow in patients’ clinical notes [24, 25].

*d) Infection control and prevention:* Ultrasound machines, cables and probes need to be cleaned and disinfected to minimize the risk of cross contamination before and after every use. Single-use sterile sachets are preferred over large gel bottles, which may become colonized with pathogens [26].

## 5) Outlining a clinical workflow

**Fig. 1:**
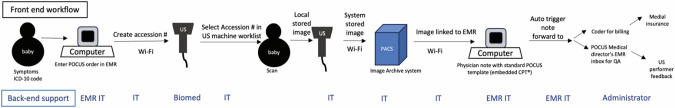
POCUS workflow example to local storage database. Biomed biomedicine, CPT current procedural terminology, EMR electronic medical record, IT information technology, PACS picture archiving and communication system, POCUS point-of-care ultrasound, QA quality assurance, US ultrasound, used with permission from Chan et al. Am J Perinatol, 2024.

Image storage and documentation are crucial aspects of building a POCUS program [27]. Compliance with the Health Insurance Portability and Accountability act (HIPAA) regulations, safeguarding patient confidentiality, and ensuring data security are pivotal features of a well-designed image storage system. Images and videos obtained during clinical scans must be accurately tagged with appropriate patient identifiers and clinical demographics. An ideal image storage pathway would allow for the ability to link/accept the order placed for a POCUS scan in the electronic medical record on the ultrasound machine prior to performing the scan. Data collected during and after the scan for that specific order will then be automatically labeled with patient identifiers and stored in hospitals’ image storage system in the patient’s record (Fig. 1). A quality assurance process should be in place to regularly review and archive stored images, ideally by divisional POCUS champions. Ensuring secure storage of images acquired by learners during training presents a unique challenge, as most hospital image archive systems are typically limited to storing images obtained during clinical scans. HIPAA compliant, secure cloud-based image storage services specific for departmental POCUS needs could be considered, however these may pose a financial burden for smaller programs. As an alternative, external drives compliant with hospital regulations could be used to store deidentified images as an alternative practice (Table 1).

**Table 1:** Pros and cons of POCUS workflow pathways.

	EMR integration with local radiology server	EMR integration with third party server	No EMR integration
Pros	- Utilize EMR order set, patient information on POCUS machine prior to study initiation - Images saved on local radiology hospital drive	- Utilize EMR order set, patient information on POCUS machine prior to study initiation - Delineation of educational versus clinical studies on server - Well built QA process and template within program	- Rapid rollout - Low institutional cost option - Ease of delineation of educational versus clinical studies without immediate integration of images in patient chart
Cons	- Time commitment by institution for EMR order set and integration into radiology server - Lack of delineation of educational versus clinical studies on server and in patient chart	- Expense for initiation and maintenance of third party server - Images live on third party server	- Heavy workload for POCUS personnel including image downloading, collating studies for QA - No direct integration with patient chart for images - Potential for error with manual input of patient information into machine

## 6) Documentation

Thorough documentation of observations and key findings from clinical scans is imperative for the long-term success of a POCUS program. Properly documented reports including  patient demographics, type of scan, indications for the scan, descriptive summary of findings and impressions, with key images that inform clinical-decision making are essential components of the patient’s medical record. This practice not only ensures compliance with legal and regulatory standards but also promotes accountability and transparency. Appropriate documentation is required for quality assurance, effective billing, coding, and reimbursement. Creating templates within electronic medical records could promote consistent documentation among different providers. Furthermore, the contributions of these reports to future education and research could be invaluable.

Overall, a well-structured image storage and documentation pathway will enhance the credibility and quality of the POCUS program ultimately leading to improved patient care [28]. Utilizing an image repository, along with robust documenting and QA process is imperative for bedside ultrasound use to ensure accurate documentation [29].

The perceived liability, or risk of litigation, is theoretically generalized from litigations encountered by obstetricians and radiologists, where misinterpretation and misdiagnosis allegations represent  a substantial portion of malpractice claims. However, there have been no reported cases of litigation related to the use of POCUS in neonatology. In contrast, there are two cases involving litigation due to the failure to use POCUS as a diagnostic tool [30].

## 7) Establishing QA and credentialing processes

In addition to hospital staff and divisional stakeholders, support from hospital leadership is crucial for program development. Their involvement is essential for resource allocation, including personnel support and machine acquisition, as well as other factors such as biomedical engineering and information technology (IT) support for image archiving and quality improvement mechanisms. Guidance from other departments within the hospital that have established QA and credentialing processes can provide an initial framework for program development, focusing on neonatal applications. Beyond structural support, shared educational expertise is invaluable, particularly when starting a program that may have limited NICU personnel available to provide education during its early stage.

## Billing for neonatal POCUS

Billing for neonatal POCUS can be complex and challenging, particularly because many NICU services are included in the daily service bundle [31, 32]. While adult specialties such as emergency care medicine and anesthesiology have established billing frameworks [2, 33, 34], there remains a lack of consensus on neonatal POCUS billing. Efficient billing adheres to standards, including specific indications for POCUS use, image acquisition, documentation, interpretation, and payment requests. Optimal imaging acquisition, labeling, and storage are essential for developing an efficient and safe neonatal POCUS service. Documentation should include details such as the provider performing the procedure, date of service, type of exam, findings, and interpretations that guide medical decisions. Automating workflows offers a promising solution to enhance compliance, productivity, and efficient reimbursement, ultimately improving patient care [35, 36]. Such workflows have demonstrated significant increases in POCUS scans, user engagement, exam compliance, quality assurance, and potential revenue. Multidisciplinary collaboration among providers, coders, billing team, and institutional IT departments is essential for developing efficient workflows and regular optimization to meet evolving demands as the neonatal POCUS services grow.

## Conclusion

The use of POCUS in neonatology is rapidly expanding, and a well-defined roadmap will help guide its development and streamline progress. The National Neonatal POCUS Collaborative was established to provide a forum for neonatal POCUS stakeholders to build consensus on programmatic development, including guideline creation, credentialing pathways, and training support. A multifaceted approach to NICU POCUS development through establishment of programmatic needs and maintenance will enable POCUS to become standard of care in the NICU, thereby enhance the care of this fragile patient population.
